# Supplementary material for: Differences in local immune cell landscape between Q fever and atherosclerotic abdominal aortic aneurysms identified by multiplex immunohistochemistry
Source: eLife. 2022 Feb 9;11:e72486. doi: 10.7554/eLife.72486 (PMC8871373; doi:10.7554/eLife.72486)
Supplement: Supplementary file 1. [file elife-72486-supp1.docx]

**Supplementary file 1**

| PCA1 | | | PCA2 | | |
| --- | --- | --- | --- | --- | --- |
|  | PC1 | PC2 |  | PC1 | PC2 |
| Percentage neutrophils | 0.1552966 | -0.95938201 | Percentage neutrophils | 516,36 | 1821,29 |
| Percentage T cells | -0.7273946 | -0.27008496 | Percentage MMP9 positive neutrophils | -163,75 | -4250,44 |
| Percentage B cells | -0.6517750 | 0.07806938 | Percentage macrophages | -693,36 | -982,44 |
| Percentage CDC2 | -0.0094728 | 0.00237974 | Percentage M1 macrophages | -469,67 | 3542,92 |
| Percentage macrophages | -0.1479181 | -0.02323317 | Percentage GMCSF positive M1 macrophages | -705,11 | 80,70 |
|  | | | Percentage M1 macrophages in infiltrate | -3412,24 | -2848,68 |
|  |  |  | Percentage MMP9 positive M1 macrophages | -207,85 | -437,87 |
|  |  |  | Percentage M1 macrophages in tissue | 3412,24 | 2848,68 |
|  |  |  | Percentage M2 macrophages | 469,67 | -3542,92 |
|  |  |  | Percentage GMCSF positive M2 macrophages | -232,48 | -301,30 |
|  |  |  | Percentage M2 macrophages in infiltrate | -313,83 | -1463,77 |
|  |  |  | Percentage MMP9 positive M2 macrophages | 119,56 | 259,98 |
|  |  |  | Percentage M2 macrophages in tissue | 313,83 | 1463,77 |
|  |  |  | Percentage MMP9 positive macrophages | -79,59 | 19,63 |
|  |  |  | GMCSF positive pixels corrected for macrophages | -86,67 | 3217,79 |
|  |  |  | Percentage T cells | -2180,58 | -3188,14 |
|  |  |  | Percentage cytotoxic T cells | 869,39 | -11045,55 |
|  |  |  | Percentage cytotoxic T cells in infiltrate | -5824,95 | -6658,77 |
|  |  |  | Cytotoxic T cells per infiltrate area | 5601,63 | -989077,50 |
|  |  |  | Percentage cytotoxic T cells in tissue | 5824,95 | 6658,77 |
|  |  |  | Percentage helper T cells | -869,39 | 11045,55 |
|  |  |  | Percentage memory T cells | 155,55 | 4093,50 |
|  |  |  | Percentage non-memory T cells | -155,55 | -4093,50 |
|  |  |  | Memory T cells per infiltrate area | -5430,45 | 609,06 |
|  |  |  | Memory T cells per tissue area | 5430,45 | -609,06 |
|  |  |  | Percentage of non-memory T cells in infiltrate | -7380,74 | -820,21 |
|  |  |  | Percentage of non-memory T cells in tissue | 7380,74 | 820,21 |
|  |  |  | Percentage regulatory T cells | -804,13 | -698,70 |
|  |  |  | Percentage regulatory T cells in infiltrate | -7218,02 | 3767,40 |
|  |  |  | Regulatory T cells per infiltrate area | -77030,30 | -77745,88 |
|  |  |  | Percentage regulatory T cells in tissue | 6580,36 | -6416,75 |
|  |  |  | Regulatory T cells per tissue area | -774,68 | -7320,17 |
|  |  |  | Percentage T cells in tissue | 7048,73 | 142,36 |
|  |  |  | T cells per tissue area | -3743,11 | -122418,90 |
|  |  |  | Percentage B cells | -3654,05 | 211,69 |
|  |  |  | Percentage B cells in infiltrate | -5632,75 | -2590,95 |
|  |  |  | B cells per infiltrate area | -996561,50 | 1180,00 |
|  |  |  | Percentage B cells in tissue | 5632,75 | 2590,95 |
|  |  |  | B cells per tissue are | -19889,18 | -10651,42 |
|  |  |  | Percentage CDc2 | -41,23 | 87,21 |

Supplementary File 1A: Loadings of Principal Component Analysis 1 and 2 as depicted in Figure 4.

|  |  | Vendor | Concentration |
| --- | --- | --- | --- |
| Primary antibodies | CD3 (SP7) | Thermo-Fisher | 1:400 |
|  | CD8 (CD8/144B) | Dako | 1:1600 |
|  | CD20 (L26) | Thermo-Fisher | 1:300 |
|  | CD1c (2F4) | Abcam | 1:150 |
|  | FoxP3 (236A/E7) | eBioscience | 1:300 |
|  | CD45RO (UCHL-1) | Thermo-Fisher | 1:3000 |
|  | CD68 (PG-M1) | Dako | 1:200 |
|  | CD206 (CL038+) | Sigma | 1:2500 |
|  | CD15 (MMA) | BD Biosciences | 1:600 |
|  | CD31 (JC70A) | Dako | 1:800 |
|  | MMP9 (polyclonal) | Atlas antibodies | 1:600 |
|  | GM-CSF (polyclonal) | Sanbio | 1:200 |
|  |  |  |  |
| Other equipment and reagents | Silane coated glass slides | New Silane III, MUTO PURE CHEMICALS, Japan |  |
|  | BOND Epitope Retrieval 1 | AR9961, Leica Biosystems |  |
|  | BOND Epitope Retrieval 2 | AR9640, Leica Biosystems |  |
|  | Akoya Antibody Diluent/Block | Akoya biosciences, MA |  |
|  | Polymer HRP Ms + Rb | Akoya biosciences, MA |  |
|  | Plus Amplification Diluent | Akoya biosciences, MA |  |
|  | Fluoromount-G | Southern Biotech, Birmingham, AL, USA |  |
|  | Opal 7-Color IHC kit including DAPI, Opal 520, Opal 540, Opal 570, Opal 620, Opal 650, Opal 690 | Perkin Elmer |  |

Supplementary File 1B: Overview of reagents and dilutions.
